# LATENT DYADIC CHANGE MODEL OF SPOUSAL SIMILARITY IN PHYSIOLOGICAL MARKERS OF AGING

**DOI:** 10.1093/geroni/igz038.3440

**Published:** 2019-11-08

**Authors:** Shannon T Mejia, Richard Gonzalez

**Affiliations:** 1 University of Illinois, Urbana-Champaign, Champaign, Illinois, United States; 2 University of Michigan, Ann Arbor, Michigan, United States

## Abstract

Older adult populations are known for their diversity in health. Within couples, however, population studies have documented exceptional levels of spousal similarity in health and health behaviors. The presence of spousal similarity in health among older adults suggests a process of convergence, yet few studies have examined this phenomenon longitudinally. We present a latent dyadic change model to estimate the extent to which couples’ similarity in grip strength, Cystatin C, and lung function--indicators of frailty/physiologic reserve. The model is a longitudinal extension of the latent dyadic model, where husbands’ and wives’ markers of health are parsed into variance that is attributed to the couple and individual levels. Change in the biomarkers of aging is then estimated at the couple and individual levels, resulting in estimates of husbands’ change, wives’ change, and shared change. We illustrate our model using physiological data from the Health and Retirement Study, a nationally representative panel study of individuals aged 51+ in the United States (3,500+ eligible couples). At the individual level, grip strength and lung function decreased, whereas Cystatin C increased for both husbands and wives. The shared change parameter estimated 16% to 25% of the change in markers of aging existed at the couple level. This suggests, consistent with convergence, that similarities in markers of aging at T2 were due to shared processes of change. Shared processes held after adjusting for indicators of partner selection. The latent dyadic change model offers a methodology to examine change in couples’ shared processes over time.

